# A197 INVESTIGATING THE PROTON-PUMP INHIBITOR USE PRECEDING AND POST AN INFLAMMATORY BOWEL DISEASE DIAGNOSIS

**DOI:** 10.1093/jcag/gwac036.197

**Published:** 2023-03-07

**Authors:** N Singh, Z Nugent, H Singh, S Shaffer, C Bernstein

**Affiliations:** 1 Internal Medicine, University of Manitoba; 2 University of Manitoba Inflammatory Bowel Disease Clinical and Research Centre; 3 CancerCare Manitoba, Winnipeg, Canada

## Abstract

**Background:**

Proton-pump inhibitors (PPI) have an impact on gut microbiome. The use of PPI has increased since the late 1980s and between 2007-2017, there were nearly 59 million PPIs dispensed annually in France.^1 ^Considering that inflammatory bowel disease (IBD) affects persons of all races, a disease trigger for IBD may be a factor that can directly alter the intestinal immune response, or indirectly through changes in the gut microbiome. This raises the possibility that the advent use of PPI may be associated with the worldwide rise in IBD.

**Purpose:**

We investigated whether increased use of PPI was associated with a diagnosis of IBD.

**Method:**

The University of Manitoba IBD Epidemiology Database includes all Manitobans diagnosed with IBD between 1984 to 2018 with age, sex and geography-matched controls and comprehensive prescription drug data from April 1995. Subjects were considered to be users if they received 2 prescriptions of PPI. We assessed PPI prescriptions pre-diagnosis and for 3-years post-diagnosis of IBD. The absolute and relative rates were calculated and compared for PPI use pre- and post-IBD diagnosis.

**Result(s):**

A retrospective analysis was completed by analyzing 5920 subjects that were diagnosed with IBD after April 1996. Rates of PPI use in controls increased gradually from 1.5 to 6.5% over 15-years. Persons with IBD have a higher rate of PPI use peaking up to 17% within 1-year of IBD diagnosis with a rate ratio (RR) of 3.1 [95% CI 2.9 – 3.3]. Furthermore, persons with Crohn’s disease (CD) [RR= 4.2; 95% CI 3.7 – 4.6] were more likely to have been PPI users pre-diagnosis than persons with ulcerative colitis [RR= 2.4; 95% CI 2.2 – 2.7]. Important predictors of increased PPI use were older age, year of data collection and CD diagnosis.

**Conclusion(s):**

This retrospective analysis showed an increase in PPI use over the past 20-years among all Manitobans. Persons with IBD have higher PPI use within 1-year of their IBD diagnosis. It is possible that persons with IBD have an increase in acid peptic diseases or alternatively, the use of a PPI alters the gut microbiome increasing the risk for IBD diagnosis, or that some IBD symptoms are treated with PPI whether warranted or not. Future studies are needed to delineate whether the indication for ongoing PPI use is appropriate.

References:

^1^Lassalle M, Le Tri T, Bardou M, et al. Use of proton pump inhibitors in adults in France: a nationwide drug utilization study. *Eur J Clin Pharmacol*. 2020;76(3):449-457.

**Please acknowledge all funding agencies by checking the applicable boxes below:**

None

**Disclosure of Interest:**

None Declared

